# Surveillance of antibiotic resistance among common *Clostridium difficile* ribotypes in Hong Kong

**DOI:** 10.1038/s41598-017-17523-7

**Published:** 2017-12-08

**Authors:** Viola C. Y. Chow, Thomas N. Y. Kwong, Erica W. M. So, Yolanda I. I. Ho, Sunny H. Wong, Raymond W. M. Lai, Raphael C. Y. Chan

**Affiliations:** 1 Department of Microbiology, Faculty of Medicine, The Chinese University of Hong Kong, Shatin, Hong Kong SAR; 2 Li Ka Shing Institute of Health Sciences, The Chinese University of Hong Kong, Shatin, Hong Kong SAR; 3 Institute of Digestive Disease, State Key Laboratory of Digestive Disease, and Department of Medicine and Therapeutics, Faculty of Medicine, The Chinese University of Hong Kong, Shatin, Hong Kong SAR; 4 The Chinese University of Hong Kong Shenzhen Research Institute, Shenzhen, China

## Abstract

Incidence of *Clostridium difficile* infection (CDI) is rapidly increasing and it poses a major health burden globally. However, data regarding the epidemiology of CDI in Asia are limited. We aimed to characterize the antimicrobial susceptibility patterns of common ribotypes of toxigenic *C. difficile* in Hong Kong. Fifty-three PCR ribotypes were identified among 284 toxigenic *C*. *difficile* clinical isolates. The five most prevalent ribotypes were 002 (13%), 017 (12%), 014 (10%), 012 (9.2%), and 020 (9.5%). All tested *C*. *difficile* strains remained susceptible to metronidazole, vancomycin, meropenem and piperacillin/tazobactam, but highly resistant to cephalosporins. Of the fluoroquinolones, highest resistance to ciprofloxacin was observed (99%), followed by levofloxacin (43%) and moxifloxacin (23%). The two newly emerged PCR ribotypes, 017 and 002, demonstrated high levels of co-resistance towards clindamycin, tetracycline, erythromycin and moxifloxacin. PCR ribotypes 017 and 002 with multi-drug resistance are rapidly emerging and continuous surveillance is important to monitor the epidemiology of *C. difficile* to prevent outbreaks of CDI.

## Introduction

*Clostridium difficile* is a Gram-positive, anaerobic, spore-forming bacillus, which is associated with various gastrointestinal manifestations ranging from mild diarrhoea to extremely severe complications, including pseudomembranous colitis and toxic megacolon^1–3^. Although the exact reason remains elusive, the incidence of *C. difficile* infection (CDI) is rapidly increasing in many countries including those in East Asia^4–10^. For instance, the rate of CDI in Korea was estimated to have increased from 1.43 per 100,000 in 2008 to 5.06 per 100,000 in 2011^9^. The significant increase in morbidity and mortality associated with CDI also poses a major health burden globally^11^. It was reported that Healthcare attributable to CDI was US$6.3 billion in U.S.^12^ and similar trends were observed in different European countries^13–15^. The most common risk factor for CDI is exposure to broad-spectrum antibiotics, especially with usage of fluoroquinolones, clindamycin and third-generation cephalosporins^16^. Other risk factors include old age, prolonged hospitalization, anti-neoplastic chemotherapy, surgery and procedures, and severe underlying systemic diseases^17,18^. Since 2003, the emergence of the hypervirulent *C. difficile* strain, restriction endonuclease analysis type BI, North American pulsed field type 1 and PCR ribotype 027 (B1/NAP1/027), has led to an increased mortality during outbreaks in Europe, Canada, and the U.S.^19,20^. While this PCR ribotype 027 strain remained prevalent in North America, surveillance reports suggested that incidence caused by this strain was decreasing in Europe, while another hypervirulent strain, PCR ribotype 078 that was first isolated from animals and food products, was emerging as the dominant clone with a strong association with community-acquired CDI^21^.

In Hong Kong, the *C. difficile* PCR ribotype 027 was first identified in 2008 but the PCR ribotype 078 strain has not been identified^22^. The PCR ribotype 002 was identified as the predominant clone in 2009,^8,23^ however, it is uncertain whether this clone has persisted, and information on the antimicrobial susceptibility of various *C. difficile* ribotypes in Hong Kong is scarce. The present study aimed to identify prevalent *C*. *difficile* ribotypes in Hong Kong and characterize their antimicrobial susceptibility patterns. Such information is valuable for better control and prevention of CDI in this region.

## Results

### Antimicrobial susceptibility patterns

In this study, 284 *C*. *difficile* isolates collected at the Prince of Wales Hospital, Hong Kong between 2009–2011 were analysed. The susceptibility patterns of the isolates to 15 antimicrobial agents were determined (Table 1). According to the CLSI criteria for antimicrobial susceptibility^24^, all isolates were susceptible to metronidazole, meropenem, and piperacillin-tazobactam, but were resistant to cefotaxime (Table 1). The resistance rates to cefoperazone, clindamycin, tetracycline and moxifloxacin were 96% (274/284), 84% (239/284), 30% (84/284) and 23% (64/284), respectively (Table 1). As determined with the suggested breakpoints for vancomycin and fusidic acid from EUCAST^25^, 4 out of 284 isolates (1.4%) were resistant to vancomycin (MIC = 4 mg/L) while their resistance rate to fusidic acid was 40% (114/284) (Table 1). Based on the breakpoints described by Huang *et al*.^26^, the resistance rates displayed by the clinical isolates to erythromycin, ciprofloxacin, levofloxacin, and rifampicin were 46% (131/284), 99% (281/284), 43% (121/284) and 10% (28/284), respectively (Table 1). The MIC range, MIC_50_ and MIC_90_ values for ceftazidime were identical as cefoperazone (Table 1), rendering this drug as ineffective against *C. difficile*, which was expected to show resistance to cefoperazone.

**Table 1:** *In vitro* susceptibilities of 284 toxigenic *C. difficile* clinical isolates to 15 antimicrobial agents in Hong Kong.

***Antibiotic***	***Range (mg/L)***	***MIC***_***50***_ ***(mg/L)***	***MIC***_***90***_ ***(mg/L)***	***Resistance breakpoint (mg/L)***	**% Resistance**
Metronidazole	≤0.125–1	≤0.125	≤0.125	32^a^	0
Vancomycin	0.5–4	1	1	>2^b^	1.4
Clindamycin	≤1–>128	16	>128	8^a^	84
Tetracycline	≤0.125–64	0.25	32	16^a^	30
Erythromycin	≤1–>128	2	>128	8^c^	46
Rifampicin	≤0.125–>8	≤0.125	2	4^c^	10
Fusidic acid	0.5–8	2	4	2^b^	40
Moxifloxacin	1–32	2	32	8^a^	23
Ciprofloxacin	2–>128	16	128	8^c^	99
Levofloxacin	2–>128	4	>128	8^c^	43
Meropenem	≤1–8	2	4	16^a^	0
Piperacillin-tazobactam	4/4–32/4	16/4	16/4	128/4^a^	0
Cefotaxime	128–>256	256	>256	64^a^	100
Cefoperazone	32–>256	64	>256	64^a^	96
Ceftazidime	32–>256	64	>256		

^a^MIC breakpoints for *C. difficile* recommended by the Clinical and Laboratory Standards Institute^24^.

^b^MIC breakpoint was based on the recommendation by the European Committee on Antimicrobial Susceptibility Testing^25^.

^c^MIC breakpoints were according to Huang *et al*.^26^.

### PCR ribotypes

**Figure 1:**
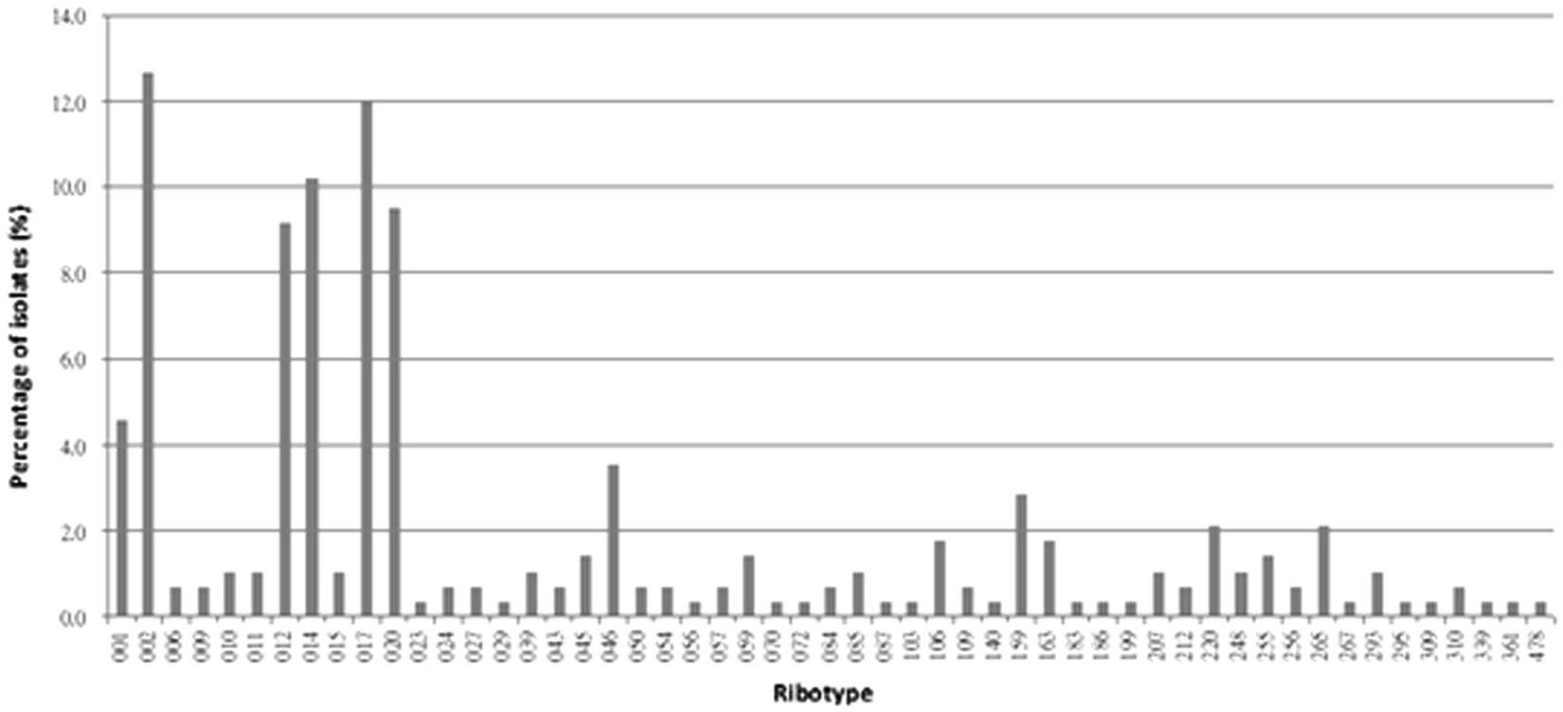
Distribution of PCR ribotypes among the 284 toxigenic *C. difficile* strains isolated in Hong Kong.

Fifty-three PCR ribotypes were identified among the 284 toxigenic *C. difficile* clinical isolates and their respective distribution frequencies are shown in Fig. 1. The predominant ribotypes isolated in Hong Kong were 002, 017, 014, 012 and 020, with frequencies of 13% (36/284), 12% (35/284), 10% (29/284), 9.2% (26/284) and 9.5% (27/284), respectively. Altogether, these five major ribotypes accounted for 54% of the total number of isolates included in this study. The less-common ribotypes included 001, 046, 159, 220 and 265 with frequencies between 2.1% and 4.5% (Fig. 1). The remaining 31% isolates (i.e. 89 out of 284) were composed of 43 different ribotypes, with frequencies of ≤1.8% for each ribotype (Fig. 1). In our *C*. *difficile* collection, only 2 isolates of the collection were identified as PCR ribotype 027, whilst ribotype 078 remained unobserved.

### Relationship of resistance profile to prevalent ribotypes

**Figure 2:**
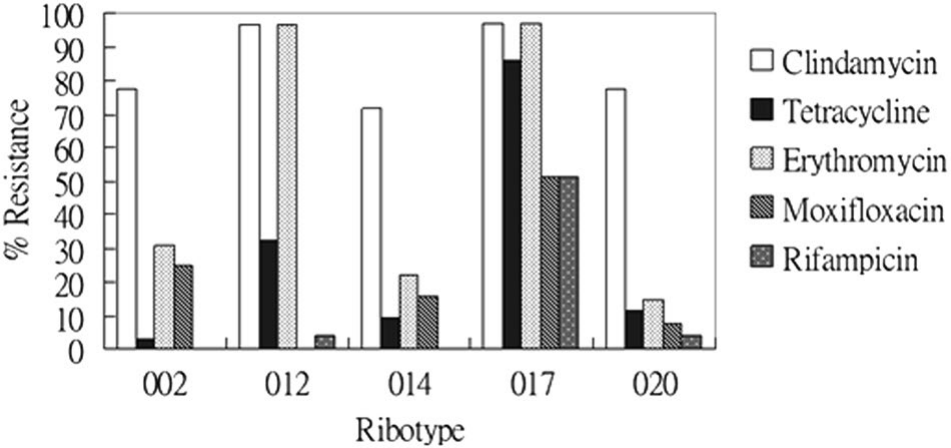
Rates of resistance to clindamycin, tetracycline, erythromycin, moxifloxacin, and rifampicin, of the five most prevalent ribotypes of toxigenic *C. difficile* clinical isolates in Hong Kong.

All *C. difficile* isolates were susceptible to metronidazole, meropenem and piperacillin-tazobactam, while all isolates were resistant to cefotaxime. The pattern of co-resistance to clindamycin, tetracycline, erythromycin, moxifloxacin, and rifampicin by the five most prevalent PCR ribotypes were investigated (Fig. 2). These five antibiotics were chosen as they represented common standalone antibiotics prescribed clinically, and showed intermediate levels of resistance. Co-resistance between clindamycin and tetracycline was most notable for ribotype 017 (97%) and ribotype 012 (96%). In addition to clindamycin and tetracycline, nearly 50% of the ribotype 017 isolates were multi-drug resistant (i.e. also resistant to moxifloxacin and rifampicin). A similar multi-drug resistance profile was observed for ribotype 020; however, the multi-drug resistance rate was much lower at only 3.7% in contrast to ribotype 017. Different patterns of co-resistance were observed for other ribotypes - isolates of both 002 and 014 ribotypes were susceptible to rifampicin, whereas all ribotype 012 stains were susceptible to moxifloxacin (Fig. 2).

## Discussion

Metronidazole has long been used as the first-line drug for treatment of CDI while vancomycin is reserved for patients with complicated infections, severe or recurrent diseases of CDI^27^. *In vitro* susceptibility testing against *C. difficile* is not routinely performed and therefore the susceptibility profiles of clinical isolates remains largely unknown. A previous study that examined 100 *C. difficile* isolates in Hong Kong identified one strain that exhibited an unexpectedly high MIC towards metronidazole (64 mg/L by E-test)^28^. Although metronidazole resistance in *C. difficile* have been described in previous reports^29,30^, our present study revealed that all *C*. *difficile* isolates were susceptible to metronidazole with MICs ranging from ≤0.125 to 1 mg/L (Table 1), which was significantly lower than the CLSI described breakpoint of 32 mg/L^24^. Thus, metronidazole should remain effective as a first-line therapy for CDI in Hong Kong. According to the EUCAST guideline^25^, four vancomycin-resistant isolates with MIC of 4 mg/L (1.4%) were identified. Isolates with reduced susceptibility to vancomycin had been described in China and Taiwan^31,32^. Nevertheless, it should be noted that the EUCAST-defined vancomycin breakpoint of >2 mg/L is only a reference point to distinguish strains with reduced susceptibility to vancomycin. In clinical settings, the faecal level of vancomycin can reach a value of ≥2000 mg/L with standard oral vancomycin treatment^33^. Therefore, despite four isolates showing reduced susceptibility to vancomycin, standard oral administration of vancomycin should remain effective for treating CDI in Hong Kong.

In the present study, most *C. difficile* isolates tested were resistant to cefotaxime, cefoperazone and ceftazidime. For quinolones, 281 of the 284 (99%) clinical isolates were resistant to ciprofloxacin, followed by levofloxacin (121/284, 43%) and moxifloxacin (65/284, 23%) (Table 1). Resistance to fluoroquinolone in *C. difficile* is mediated through changes in *gyrA* or *gyrB*, in which single mutations may raise the MIC and produced a level of resistance above peak drug concentrations achievable in serum^34^. Given the different potencies of fluoroquinolones in targeting DNA gyrase in *C. difficile*, mutations affecting the enzyme targets may confer resistance to different extents. Other factors affecting drug resistance to fluoroquinolones may include drug permeation and presence of an efflux system^35^, although the latter has not yet been demonstrated for *C. difficile*.

High resistance rate to clindamycin was also observed in our collection (239/284, 84%; Table 1). Resistance to erythromycin, tetracycline and fusidic acid were observed at 46%, 30% and 40%, respectively (Table 1). Mutations at the erythromycin ribosomal methylases gene class B (*ermB*) is a predominant mechanism of resistance to the macrolide–lincosamide–streptogramin B (MLS_B_) family of antibiotics. Nevertheless, several *ermB*-negative strains resistant to both erythromycin and clindamycin, or only to erythromycin have been identified^36–39^, suggesting presence of other resistance mechanisms. Alterations in the 23 S rDNA or ribosomal proteins (L4 or L22) have been found in some of these strains^39^, whereas the multidrug resistance (MDR) gene *cfr* have also been suggested to cause resistance to the MLS_B_ family of antibiotics^40^. Furthermore, the antibiotics may also induce *ermB* differentially, resulting in the heterogeneity of antibiotic resistance^41^. These may have accounted for the different resistance rates to clindamycin and erythromycin as observed in some studies^36,42^. Given the high levels of antibiotic resistance, prescription of antibiotics must be justified to minimize the risk of secondary infections such as CDI.

Rifampicin is an anti-tuberculosis drug and the reported resistance rates of *C. difficile* were 3.8% in Sweden, 7.9% in North America, and 25% in Shanghai^2,26,43^. The resistance rate in our study was 10% (Table 1). Nevertheless, out of the 26 rifampicin-resistant isolates, 15 of them (58%) belonged to ribotype 017. Given the large number of Chinese population being affected by tuberculosis, the high resistance rate to rifampicin in China could be a result of selective pressure exerted by the widespread use of this drug^44–46^. Since ribotype 017 was identified as the dominant clone in Shanghai^43^, the subsequent emergence of ribotype 017 in Hong Kong suggested that there might have been a clonal spread of this ribotype across the region.

Different *C. difficile* PCR ribotypes were found circulating in Hong Kong during the study period, as a total of 53 PCR ribotypes was identified. Previous study had shown that the PCR ribotype 002, with a frequency of 10%, was the dominant strain in Hong Kong in 2009, and the frequencies for ribotypes 012, 014, 017 and 020 were 2.3%, 1.2%, 0.6%, and 0%, respectively^22^. Our study confirmed that ribotype 002 remained as the predominant clone in Hong Kong (13%). However, PCR ribotype 017, with a distribution frequency of 12%, has become the second most prevalent ribotype. Other PCR ribotypes including 012, 014, and 020 were also identified as major clones at frequencies of 9.2%, 10% and 9.5%, respectively (Fig. 1). The differences between our findings and those of Cheng *et al*.^23^ suggested that the epidemiology of *C. difficile* in Hong Kong has constantly been changing. The increased sporulation rate of ribotype 002 might render this ribotype with a better survival, which might be contributing factor for their increasing prevalence^22,47^. This may also be related to the local antibiotic usage, as antibiotic prescriptions were observed to correlate highly with incidence of *C difficile* infections^48,49^.

Consider the heavy flow of international trading and long survival of *C*. *difficile* spores, it has been speculated that PCR ribotypes 012, 014 and 020 might have spread from European countries to Hong Kong, as these ribotypes has been described to cause major epidemics in Europe^50,51^. Ribotype 027 was identified as a hypervirulent strain, responsible for severe outbreaks in North America and Europe^52,53^, while ribotype 078 has emerged as another hypervirulent strain in the Netherlands^54^. Ribotype 027 arrived in Hong Kong in 2008^22^ but ribotype 078 has not yet been identified. Nonetheless, repeated outbreaks associated with the PCR ribotype 027 has not been reported in Hong Kong and its incidence rate remained low. In North America, multi-drug resistance (i.e. to clindamycin, moxifloxacin, and rifampicin) was frequently associated with ribotype 027 and was also observed among several isolates of ribotype 017^2^. Interestingly, the two ribotype 027 isolates identified in this study did not show multi-drug resistance. Among the five major ribotypes identified in this study, the rates of concurrent resistance to clindamycin, tetracycline, erythromycin, moxifloxacin and rifampicin were the highest for ribotype 017 (Fig. 2). An association between multi-drug resistance and ribotype 017 has also been reported in Poland, Korea and Shanghai^26,55,56^. A high -level of clindamycin and erythromycin co-resistance was displayed by ribotype 012 (Fig. 2). However, in contrast to ribotype 012 strains isolated in Sweden that had high resistance rates to moxifloxacin and rifampicin^57^, the ribotype 012 isolates from Hong Kong were all susceptible to moxifloxacin and largely (96%) susceptible to rifampicin. A recent surveillance report showed that ribotype 002 remained as the most prevalent strain in Hong Kong, despite the lower multi-drug resistance rate (Fig. 2)^8^. All together, these results indicated that even the same ribotype from different regions could display significantly different levels of virulence and patterns of antibiotic resistance. This may imply that environmental factors can pose a strong evolutionary pressure for their survival, and further shape their genomes in their resistance to antibiotics.

This study showed that metronidazole and vancomycin remain effective for the treatment of infection caused by toxigenic strains of *C. difficile* in Hong Kong. Ribotype 002 was identified as the most prevalent ribotype, with a high rate of co-resistance between clindamycin and erythromycin. Ribotype 017 was the second major clone in our study and is associated with multi-drug resistance. Although metronidazole and vancomycin remain effective for CDI treatment, PCR ribotypes 002 and 017 with multi-drug resistant patterns are rapidly emerging. These data inform the susceptibility patterns of these regionally prevalent ribotypes, and emphasize the need for continual surveillance on the disease.

## Methods

### Bacterial isolates

A total of 284 non-duplicate toxigenic clinical isolates of *C. difficile*, identified between December 2009 and December 2011 by the Microbiology laboratory of the Prince of Wales Hospital of Hong Kong, were included in this study. These isolates were recovered and stored in 10% glycerol broth medium at −80 °C.

### Growth conditions and cytotoxicity assay

*Vero* cell line (ATCC CCL-81) was maintained in minimum essential medium (MEM) containing 10% fetal calf serum (Gibco^®^) and gentamicin (Rotexmedica) at a final concentration of 24 mg/L. *C. difficile* isolates were maintained on anaerobic blood agar plate, supplemented with vitamin K1 (Oxoid), and grown in pre-reduced brain heart infusion (BHI) broth (Oxoid) anaerobically at 37 °C. All *C*. *difficile* isolates were confirmed as toxigenic by testing for the presence of toxin B in culture supernatant with the *C. difficile* Toxin/Antitoxin Kit (Techlab) by following the manufacturer’s instructions. In brief, each well of a 96-well plate (Greiner) was seeded with 200 μL of Vero cell culture and incubated for 24 h at 37 °C with 5% CO_2_ to achieve a confluent homogenous monolayer. Grown *C*. *difficile* broth culture was centrifuged at 6000 × *g* for 5 min and the resulting supernatant was filtered through a membrane filter with a pore size of 0.45 μm (Millipore). Each filtered supernatant was serially diluted and added to the grown Vero cells. A positive cytotoxic reaction was noted by rounding of the Vero cells observed by light microscopy after 48 h of incubation at 37 °C with 5% CO_2_. Neutralization of cytotoxic effect by the *C. difficile* antitoxin confirmed the presence of toxin B in the supernatant.

### Antimicrobial susceptibility testing

The susceptibilities of the 284 toxigenic *C. difficile* clinical isolates to 15 antimicrobial agents were determined by the agar dilution method described by the Clinical and Laboratory Standards Institute (CLSI)^24^. The antimicrobial agents tested include cefotaxime, cefoperazone, ceftazidime (GlaxoSmithKline), ciprofloxacin, clindamycin, erythromycin, fusidic acid, levofloxacin, metronidazole (B. Braun Medical Industries), meropenem (Astra Zeneca), moxifloxacin (Bayer), piperacillin-tazobactam, rifampicin, tetracycline and vancomycin, all reagents were purchased from Sigma unless otherwise stated. *C. difficile* ATCC 700057 and *Bacteroides fragilis* ATCC 25285 were used as control strains for each run of agar dilution testing. The minimal inhibitory concentration (MIC) was defined as the lowest concentration of the drug that inhibits bacterial growth. The breakpoints for metronidazole, clindamycin, tetracycline, moxifloxacin, meropenem, piperacillin-tazobactam, cefotaxime and cefoperazone were determined with MIC criteria described by CLSI guidelines^24^. For vancomycin and fusidic acid, breakpoints recommended by the European Committee on Antimicrobial Susceptibility Testing (EUCAST) were used^25^. For erythromycin, ciprofloxacin, levofloxacin, and rifampicin, we adapted the breakpoints from Huang *et al*.^26^. No breakpoint for ceftazidime was available at the time of this study.

### PCR ribotyping

PCR ribotyping was performed as previously described^58^. In brief, crude template nucleic acid was prepared by resuspending *C*. *difficile* cells, which were grown on Anaerobe Agar (LabM) supplemented with 6% horse blood, in a 5% (wt/vol) solution of Chelex-100 (Bio-Rad) and boiling. After removal of cellular debris by centrifugation, the resulting supernatant (10% vol/vol) was added to PCR mixture containing 50 pmol of each primer, 5′-CTGGGGTGAAGTCGTAACAAGG-3′ (positions 1445 to 1466 of the 16 S rRNA gene) and 5′-GCGCCCTTTGTAGCTTGACC-3′ (positions 20 to 1 of the 23 S rRNA gene). Reaction mixtures were subjected to 35 cycles of denaturation at 94 °C for 1 min, annealing at 55 °C for 1 min, and extension at 72 °C for 2 min. Amplification products were separated by electrophoresis in 3% Metaphor agarose. Amplified products were visualized by ethidium bromide staining and the ribotype patterns were analyzed with image analysis software.
